# Collectanea Medica, Consisting of Anecdotes, Facts, Extracts, Illustrations, Queries, Suggestions, &c.

**Published:** 1819-05

**Authors:** 


### COLLECTANEA MEDICA,

CONSISTING OF

### ANECDOTES, FACTS, EXTRACTS, ILLUSTRATIONS, QUERIES, SUGGESTIONS, &c.

RELATING TO THE

*History or the Art of Medicine, and the Auxiliary Sciences.*

Quicquid agunt medici, nostri farrago libelli.

### *Anatomico-Physiological Examination of the Medicinal Leech;* by HENRY LEBRECHT KURZMANN, M.D. Physician to his Majesty the King of Prussia, &c.

(*Continued from p.* 314.)

AT the upper rim of the œsophagus, or rather more upon that part of the inner surface of the general muscular coat just below it, three rows of teeth are found standing at equal distances from, and forming an angle of 120 degrees to, each other. Each row has two skinny parietes, or lateral surfaces, which, from a broad basis in front, join each other over a sharp dented arch in such a manner that the points of that arch pass through them. The inner space between these parietes is filled with minute fleshy particles, probably constituting the muscles requisite for moving these rows of teeth. In order to obtain a clear view of the shape of these teeth, an incision must first be made with a sharp knife through the skin that covers them, along and at the side of the sharp edge on each side, down to the general muscular coat; then they are to be separated from the body by a transverse incision at the bottom of the row; then, without either squeezing or distorting it, the row of teeth thus separated is to be placed on a plate of glass, which, on being fixed under a microscope, must be exposed to a strong light from below. In young leeches, not yet arrived at their full growth, these teeth are merely small points fixed on the convex rim of the arch, and somewhat bent, corresponding with the convexity of the same. In old and full-grown leeches, they stand in the arch as single teeth, forming one whole in conjunction with it. Each tooth consists of a straight cylinder, with a blunt point, constituting about a fourth part of the whole length of the tooth, projecting over the convex margin of the arch. Sixty or seventy such teeth are always found in each arch. As for the rest, Dr. K. could never observe in them, even in a dry state, such a degree of hardness that could enable them to wound the skin; for which purpose they are likewise too small and slender.

Immediately behind the œsophagus the alimentary canal commences, and consists of the simple white coat that lines the inner surface of the œsophagus. In order to obtain a plain aspect of the same, a leech, which is neither too old nor too thick and void of blood, should be selected, and, after being stretched out to its utmost length, fastened with pins upon a plate of wax at its upper and lower end. This done, it should be cautiously opened along the back, so that its flapping inwards be prevented, by laterally bending the skin outwards directly as it is opened, and fastening it with pins from one half inch to the other. In this manner, a view of the whole uninjured alimentary canal, running in a straight direction from the œsophagus to the caudal part, is obtained, and found to consist of the stomach, the two coeca, and the rectum.

The stomach occupies two-thirds of the whole length of the animal, and consists of nine divisions lying one behind the other, each of them having an appendage on each side, running like a small tapering canal towards the sides, losing itself at the abdominal part into a number of fine threads, which then consolidate with the external coat. Between each two divisions, the stomach contracts itself in such a manner as to leave only a round opening of not quite a line in diameter, exactly fitting the opening of the next contraction; thus forming a straight passage from the œsophagus to the place where the coeca branch off. On the belly-side, the stomach is every where consolidated with the subjacent parts, by which its examination from the side is rendered more difficult than from the back.

From the last division of the stomach a canal, a line in length, issues forth, from which a coecum branches off on each side; which, making first a lateral bend of three or four lines in length, soon join each other inseparably, and run, with more or less considerable distention, down to the caudal end of the animal.

Towards the back, in the hollow between the coeca, the rectum has its situation. When the leech contracts itself, the under part of one division of the stomach touches the upper part of the succeeding one at that point where the opening exists between them; so that nine successive divisions are formed, running along the body, separated by thin partitions, with an opening in the centre, giving the whole a cellular appearance. This may be plainly exhibited by inflating a dead leech, by means of a tube introduced into its mouth, then drying the same, and afterwards opening it lengthways on the side with a sharp knife. The rectum, which already distinguishes itself from the white skin of the intestinal canal by its brownish colour, consists of a somewhat stronger membrane than the stomach, and, in the extended state of the animal, runs in a straight, but in the contracted one in a serpentine, direction, in the middle of the body, partly between and partly over the two coeca, towards the anal orifice. At its commencement appears on each side a strong hollow knob, the cavity of which, on account of its thick skin, is but small. The inner surface of the rectum, full of small plaits, running in a slanting circular direction, thickens at the distance of some lines from the anus, seeming to form a sphincter; then enlarges again, and presents a smooth ligamentous tube. It is always filled with a blackish-green fluid, which, on pressure only, issues from the anus. Immediately below the place where the coeca issue from the stomach, a canal makes its appearance between them, tapering at the lower end, being three lines long in its greatest dimension, and inserted between the two knobs of the rectum. On the inner surface of the same, single muscular fibres are found running lengthways, approaching closer to each other towards the rectum, and consolidating between the two knobs. Single threads turn sideways, and join the inner surface of the knobs; the skin of the canal passing over the outer surface, joins the rectum. The adhesion of the muscular fibres in this canal is so close, that it is impervious even to the air; so that, indeed, a connexion, but by no means a communication, exists between the stomach and the rectum. The separation of the excrements from the alimentary matter seems thus, by the total want of all other organs, and that of a proper communication with the stomach, probably to take place in the above-described canal, and in the two knobs of the rectum. The smallness of this organ in proportion to the largeness of the stomach, and the quantity of alimentary matter contained in it, doubtless contributes a great deal towards the exceeding slowness of the digestive process in this animal. A leech, thoroughly filled by suction, still contains blood in its stomach after a lapse of two years and a half, which has still the same slimy consistence as shortly after suction. This sort of leeches, however, lives (according to our author's observation) exclusively on blood; whereas, the horse-leech, or hirudo-sanguisuga, lives on worms and dead animals macerated by water.

In the act of sucking, the leech proceeds in the following manner:—with its upper-lip far distended in the shape of a spoon, which is enlarged by the under-lip extended latitudinally, it drops upon the spot to be sucked, closely pressing its rim all around; then, contracting its body to the utmost, it fills up the space confined by the rim of the lip, with the inner surface of the oral cavity, in particular with the two plaits, forcing the little air still left beneath the lips back into the body. This done, the animal raises the fore-part of the body a little, as if it was going to let loose, yet remaining hanging firmly by the lips; a vacuum arises, into which the skin is forced; then, inflicting a triangular wound with its teeth, it completes the operation of the cupping-glass. Whilst the blood is flowing, the leech remains quite immoveable; only in the region of the œsophagus a sucking motion is perceived, propagating itself through the whole body, by which the blood is forced into the hindmost division of the alimentary canal. In this manner a full-grown leech, void of blood, can take about three drachms and a half at the utmost.

The leech is unquestionably hermaphrodite, uniting in itself both parts of generation, and requiring a reciprocal fecundation. These parts are seen plainest on opening the animal at the back, and removing the stomach, beneath which they are situated.

The male generative organ, or penis, that opens on the abdominal parts between the 24th and 25th ring, may be divided into the sheath and the proper penis. On dissection, the sheath alone is perceivable, consisting of a globular and a cylindrical part, the whole of which has not quite two lines in length. The globular part of the sheath consists of a smooth strong white membrane, easily divisible by the knife into laminæ; on the outer side divers small blood-vessels may be observed, and on the inner one strong muscular fibres closely adhering to it, and running in a straight direction from the fore-part backwards, ending near the cylindrical part. The back-end of the globular part is continued into the thin cylindrical one of the sheath, which latter consists of a pretty firm though simple membrane, a line long, running in a straight direction backwards, bending at the lower part, and running again forward under and close to the descending part, and opening at the belly-side of the animal. At the inner rim of the opening of this cylindrical part, it is surrounded by a second very slender tube, consisting apparently of a single, though very firm and almost tendinous, membrane, which is the proper penis. It runs on backward in its sheath, finely winding in a serpentine manner in the cylindrical and the globular parts; in the latter of which it forms, besides, divers large windings, terminating in a round, white, firm, and almost cartilaginous substance, consolidated with the fundus of the globular part of the sheath. Thus, the penis is consolidated at both ends with its sheath, but, from its serpentine and bending situation, has a far more considerable length than the latter, over which it, on issuing, projects double the length: it is capable of a considerable degree of extension, perhaps of an inch and more, and tears with a cracking sound. If, in a leech opened alive, this sheath is irritated by the point of the knife, it raises itself without sensibly altering its bend, and shows considerable elasticity on being touched with the knife; but never did Dr. K. observe any projection of the penis, as is the case in the horse-leech. When, however, this organ projects from the body, which commonly happens a few days before the expiration of leeches that die in confinement towards the close of the winter, the same elasticity is observed.

On projection, the penis is inverted, so that the inner surface of the cylinder becomes the outer one. When a leech is killed with hot water, the penis projects. On opening the leech at the back, and cutting the globular part of the sheath lengthways, and then pulling the cartilaginous substance in the fundus of the same, the penis may be pulled back into the body, and may be plainly seen to turn back again. Close to this organ lies, on each side, a white yellowish substance, the left of which is larger than the right one: these probably constitute the testicles. On each of them a convex and a concave rim is discernible; the former being turned towards the penis, and the latter, which has more or less incisions, turned outwards. Each testicle ends in front in a broad basis, and backwards in a point. On the basis a canal is distinctly observed, which, originating in the concave rim, runs over its base; then proceeds beneath the globular part of the sheath, enters into its under surface in front, and loses itself in the round cartilaginous part on the fundus, sideways, forward, and upwards, from that point where the penis joins it. The skin of this canal, which is the spermatic duct, is very strong, and may be divided in layers. The testicles are covered with a very thin skin, on which, by means of a magnifying glass, blood-vessels may be discerned. In their interior are several windings of a pretty thick vessel, filled with a thickish white fluid, difficultly mixible with water. This vessel ends on one side in the funiculus spermaticus, and on the other, from the point of the testicles, it runs backwards into a fine vessel, consisting of a very fine white skin, continuing on each side of the body till about the region of the 75th ring. Along each of these vessels there lie inwardly, at an equal distance, nine roundish white substances, as big as a pin's head, each of them connected with the principal vessel by means of a short one, of an equal condition and strength; and it is only in the last of these substances that the principal vessel itself does enter directly. These substances consist of a moderately strong skin, through which windings, like those of the testicles, faintly appear; which make it probable that the thickish fluid they contain is, like that in the former, enclosed in a particular vessel. This fluid mostly has, during the winter, summer, and autumn, a white thickish consistence; but, in the spring, a more brownish and watery one. If these small substances, with their common canal, are to be considered as a continuation of the testicles, each of the latter takes up almost two-thirds of the whole length of the animal. In little leeches, not yet arrived at their full growth, the penis is of an equal proportion with that of full-grown ones; but the testicles and the nine pair of roundish substances are proportionably smaller, and the latter are of a red appearance. The uterus is quite isolated, unconnected with any other viscus: its opening appears on the belly side, between the 29th and 30th ring. From this opening a skinny, pretty wide, canal, runs on in front, which, enlarging into a proportionably large bag that passes upwards in front, bends a little to the right, gradually growing broader, but contracting itself again a little at the bottom, and thus forming the proper uterus. At its fundus an appendage is found on each side, that on the left being larger than that on the right, forming a closed small cylinder, lying in a double bend on the fundus. The right one is but a roundish knob. The inner surface of the bag is covered with visible, yet very small, muscular fibres, that run from the fundus and the appendages along the inner surface, and lose themselves in the opening. In its cavity a milky fluid is found, which is easily miscible with water; thus distinguishing itself from that contained in the testicles. In not full-grown leeches, this bag is in proportion exceedingly small.

*(To be continued.)*

### *Collection of rare and interesting Cases in Anatomy and Physiology.*

*(Continued from p.* 311.*)*

*Super-Fœtation.*—PLINY, the naturalist, relates an instance of a female slave who produced twins, one of which resembled the master of the slave, the other another man with whom she had had sexual connexion. This fact, although it might lead to the suspicion of the possibility of *super-fœtation,* still it would by no means demonstrate it: that quoted by BUFFON is more conclusive. In the year 1714, a woman of South-Carolina, when in bed, was approached by a negro, who, with a poignard in his hand, obliged her, by violent menaces, to submit to his sexual desires: nine months afterwards she was delivered of two children, one of them black, the other a mulatto. This being at that period a singular case in history, might be considered as doubtful; but a similar instance has since occurred, to support it, and apparently to prove the possibility of super-fœtation. M. DELMAS, a surgeon resident at Rouen, relates that a woman of that city, thirty-six years of age, was delivered at the hospital at Rouen, on the 26th of February, 1806, of two male children, one white, the other mulatto. This woman had been pregnant eight months; the two placentas, united in the manner generally observed in the case of twins, were expelled within a few minutes after the birth of the fœtuses. The woman lived with a white man, but she had twice had connexion with a negro about the time when she believed herself to have been pregnant four months. The infants lived but three hours. A woman of Arles produced, on November 11, 1796, a female infant, apparently at the full period of gestation, which lived seven months. The lochiæ were suppressed on the fourth day, and no milk appeared in the breasts, although measures were taken to solicit it, from the desire of the mother to suckle her child. Six weeks after her delivery, this woman, with much surprise, felt the motions of a fœtus in the uterus; but, recollecting that she had cohabited with her husband four days after her accouchement, she considered that she was pregnant from that period; but this conjecture was found to be incorrect, by her giving birth to another female child, of the full growth and developement, at the termination of the fifth month from that of the former. The milk now appeared in her breasts, with which the mother suckled both her children. This fact is attested by two medical practitioners.

BAUDÉLOCQUE relates, in the second volume of his Traité de l'Art des Accouchemens, a case of super-fœtation observed, in 1780, by Doctor DESGRANGES, of Lyons. On the birth of the first infant, the discharge from the uterus ceased immediately after delivery; no milk appeared in the breasts; and the belly remained more voluminous than ordinary. Twenty days afterwards this woman admitted the caresses of her husband, and a few days afterwards she felt the motions of a fœtus in the uterus. After the lapse of a hundred and sixty-eight days from her former accouchement, she gave birth to a full-grown infant; so that it is apparent that this was conceived about seventy-two days after the former. Both of the children were living in 1782.

An instance is related, in the *Medical Museum* of Philadelphia, of a white servant giving birth at the same time to two infants,—one white, the other black. The author, no doubt, meant to state, that the latter was a mulatto; because, if it had been a black child, it should be considered as a case of disease of the skin, an instance of monstrosity, not the offspring of a negro.

These facts, and many others that our limits oblige us to pass over in silence, seem to prove that super-fœtation, although a rare, is yet a real phenomenon. Those physiologists who still deny its possibility endeavour to account for births occurring at periods too remote to admit of their being referred to the same period of conception, by asserting that it can only be explained by the existence of a double uterus, or by a division of that organ into two cavities; circumstances which anatomical investigation has shown to take place. Those who believe that super-fœtation may happen in the ordinary state of the uterus, may support their opinion by the case of the woman of Arles, in which the two placentas were united together by their broad surfaces, which could not have happened had they not been formed in the same matrix. The cessation of the lochiæ, which was observed in this as well as in that related by Baudélocque, tends to prove that the two fœtuses were contained in the same cavity.

We refer our readers to the twenty-second, twenty-third, and twenty-fourth, volumes of the London Medical and Physical Journal for some further observations and reflections on this subject.

---

*Admonitory Reflections on the Manners of a Physician.* [From the Περὶ Εὐσχημοσύνης of Hippocrates.]

Be affable; for, if austerity is repulsive to those who are in health, it is still more so to those who are suffering from disease. Do not amuse yourself with long discourses before ill-informed people, but speak of those things with precision which are strictly necessary to their welfare. Before you visit sick persons, remember what is to be done: it is comfort and alleviation of their afflictions which they require, not verbal arguments. On entering their chamber, do not neglect either the manner in which you take your seat by them, the arrangement of your dress, or your personal actions and behaviour. Remember that you are invested with a real authority. Let your answers be short and precise. Maintain a state of calmness amidst the trouble with which you are surrounded; and be ever ready for prompt and judicious exertion of your aid. In serious cases, be diligent in your attendance, that you may witness the important changes of the disease, and promptly remedy any errors which may have recently occurred.

